# Efficacy and Safety of Laughter Yoga in Cancer Patients: A Scoping Review of Randomized Controlled Trials

**DOI:** 10.7759/cureus.59163

**Published:** 2024-04-27

**Authors:** Selvaraj Giridharan, Jawaher Ansari

**Affiliations:** 1 Oncology, Tawam Hospials, Al Ain, ARE; 2 Oncology, Tawam Hospital, Al AIn, ARE

**Keywords:** review, complementary and alternative medicine (cam), health-related quality of life (hrqol), cancer care, laughter yoga

## Abstract

The use of laughter yoga as a complementary and alternative medicine (CAM) strategy has recently gained interest as a potential supportive intervention for cancer patients. In this review, we aimed to assess the impact of laughter yoga on the quality of life of cancer patients, with a focus on evidence from randomized controlled trials (RCTs). Our analysis indicates that laughter yoga can significantly improve the quality of life of cancer patients by improving emotional and physical functioning and reducing symptoms of depression and stress. These findings suggest that laughter yoga is a promising CAM practice for enhancing cancer patients' psychological and physical health. Future research should aim to extend these studies to more extensive and more diverse populations to validate and expand upon these findings.

## Introduction and background

Cancer remains a major global health problem, significantly contributing to the burden of disease worldwide. In 2019, there were 23.6 million new cases of cancer and approximately 10 million cancer-related deaths reported across 204 countries and territories [1]. The total number of cancer cases has increased by 26.3% since 2010, while cancer deaths have risen by 20.9% during the same period, highlighting the ongoing impact of cancer on global mortality and morbidity [2,3]. Effective management strategies are of utmost importance in light of these statistics.

Significant advancements in medical treatments for cancer patients have been made in recent decades, leading to improvements in life expectancy [4]. Early detection, disease-directed clinical trials, and the development of multimodal treatments, including targeted therapies and immune checkpoint inhibitors, have collectively increased survival rates [5, 6]. For instance, the cure rate for childhood cancers has risen from about 20% in the 1960s to over 80% today [7]. Despite these advancements, cancer remains a formidable challenge, not only in terms of patient survival but also in the socioeconomic impacts associated with the disease.

Improvements in cancer survival rates have led to greater attention being given to the long-term physical and psychological challenges faced by cancer survivors. These challenges are often exacerbated by the side effects of the disease and its treatment, necessitating the need for comprehensive care strategies beyond conventional treatments [8,9]. Complementary and alternative medicine (CAM) practices, including dietary interventions, art therapy, acupuncture, massage, and exercise, are widely used by cancer survivors seeking holistic care approaches to alleviate symptoms, improve quality of life, and manage stress [10,11].

Among the many CAM practices, laughter yoga has emerged as a popular and modern exercise involving voluntary laughter, which is believed to offer similar physiological and psychological benefits as spontaneous laughter. This approach combines unconditional laughter with yogic breathing (Pranayama), potentially alleviating physical pain and psychological stress. Laughter yoga, conceptualized by Dr. Kataria in 1995 in India, also incorporates mild physical exercises, deep breathing, childlike playfulness, and laughter exercises [12]. These activities enhance mental well-being, strengthen immune systems, improve social connections, and boost self-confidence.

The potential health benefits of laughter yoga extend to reducing stress, depression, psychosomatic disorders, and pain while enhancing emotional catharsis, respiration, and blood circulation. There has been an increasing number of randomized clinical trials looking at the effect of laughter yoga on different medical conditions; our search has identified there have been 25 RCTs published over the last ten years looking at a variety of medical conditions like anxiety, depression, stress, weight control, diabetes and cancer among others [13-20]. Despite its popularity and anecdotal benefits, the scientific community continues to debate the efficacy and safety of laughter yoga for cancer patients, primarily due to variations in study designs and methodological rigor.

## Review

Objective

The review aims to evaluate the impact of laughter yoga on the quality of life of cancer patients, focusing on randomized controlled trials (RCTs). It recognizes the need for a comprehensive synthesis of available data and seeks to provide a clearer understanding of laughter yoga's role in the holistic care of cancer patients. The review will address the therapeutic benefits and limitations of laughter yoga for cancer patients by analyzing the evidence from these trials.

Methodology

A literature search was conducted across multiple databases, including PubMed, Scopus, and Cochrane Library, using relevant keywords and Medical Subject Headings (MeSH) terms to identify randomized controlled trials that examined the effects of laughter yoga on cancer patients' quality of life. Studies published between January 2000 and the present were included, while non-randomized trials, observational studies, case reports, and reviews were excluded. Extracted data included author details, year of publication, study location, sample size, cancer type, intervention and control conditions, outcomes measured, and key findings.

Results

**Figure 1:**
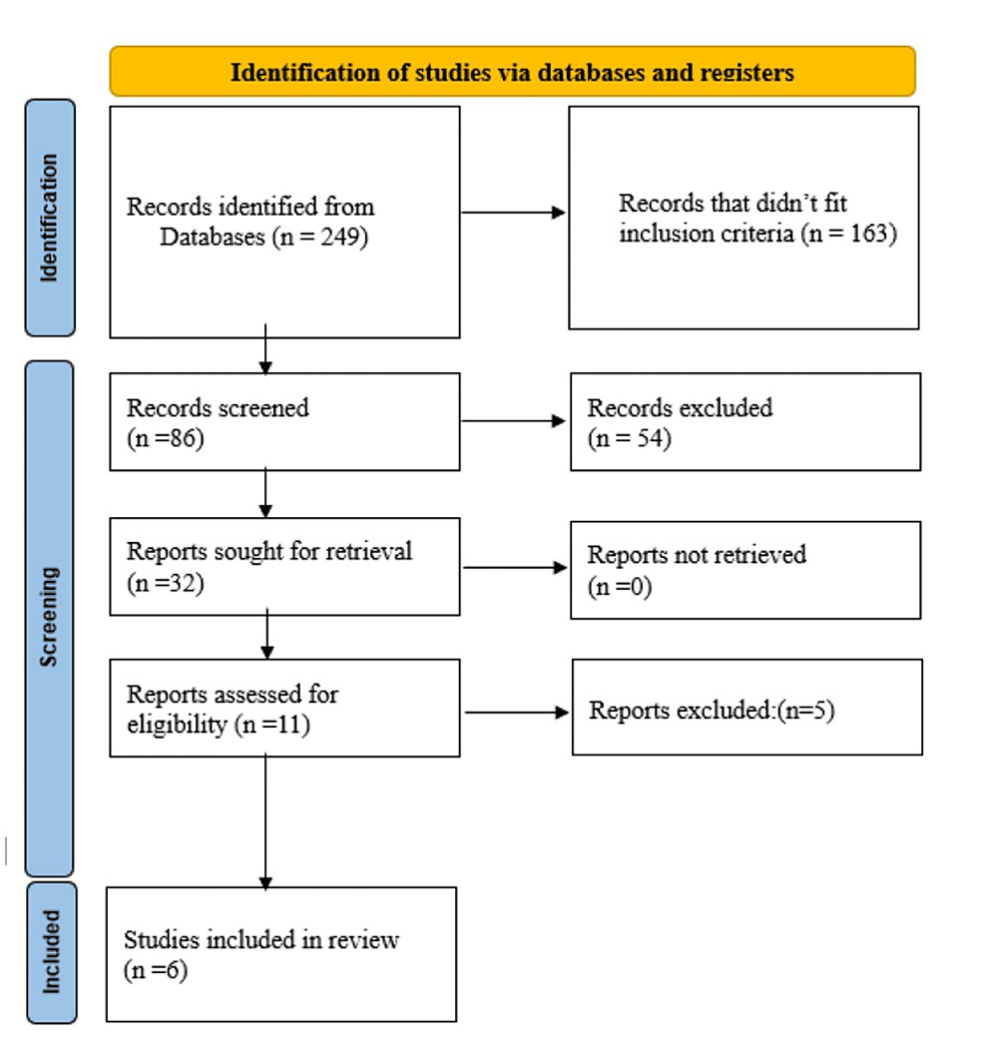
Summarized search strategy (Preferred Reporting Items for Systematic Reviews and Meta-Analysis)

A total of 249 records were identified through a comprehensive search across multiple electronic databases. After removing duplicates, 86 records remained. We then screened these records' titles and abstracts, narrowing the field to 32 potentially relevant studies based on our inclusion criteria. Further detailed assessment of these 32 studies led to the retrieval of complete texts for 11 studies, which were closely examined. Ultimately, six studies met all our inclusion criteria for detailed review and analysis (Figure 1).

Exclusions at this final stage were primarily due to non-randomized study designs, incomplete data, and studies where laughter yoga was not the primary intervention. Table 1 shows a summary of the study characteristics and the main findings. 

**Table 1:** Summary of study characteristics and main findings HRQoL - health-related quality of life; ANOVA - analysis of variance

Author and year	Country	Sample size	Study aim	Cancer type	Intervention details	Statistical tool	Conclusion
Namazania et al. 2019 [21]	Iran	69	Mental well-being	Various	4 sessions prior to chemotherapy, 20-30 min each	ANOVA, t-tests	Significant improvement in mental well-being
Mohajer et al. 2022 [22]	Iran	69	Depression	Various	Daily sessions for 4 days, 20-30 min each	t-tests	Significant reduction in depression
Namazania et al. 2023 [23]	Iran	69	HRQOL domains (physical, emotional, fatigue, etc.)	Various	4 sessions over 4 weeks, 20-30 min each	t-tests, Wilcoxon	Significant improvements in HRQOL domains
Han et al. 2023 [24]	China	84	Stress, psychological capital, exercise capacity	Lung	Intervention period not specified, sessions before assessments	t-tests, Cohen's d	Significant improvements in stress, psychological capital, exercise capacity
Morishima et al. 2019 [25]	Japan	56	pain and cognitive function	Various	Four sessions every two weeks, plus traditional comedy	Mixed-effects models	Significant improvements in pain and cognitive function
Farifteh et al. 2014 [26]	Iran	37	Stress before Chemotherapy	Various	Intervention details not fully specified	Covariance analysis	Significant reduction in stress

Upon detailed examination, we identified that three of these studies, conducted by Namazania et al. and Mohajer et al., originated from the same research group in Iran, involving the same cohort of cancer patients assessing different health outcomes [21-23]. This discovery necessitates treating these as a single study with multiple outcome measures rather than as independent observations. This study was a randomized clinical trial conducted in 2018 at the Reza Radiotherapy and Oncology Center in Iran that aimed to evaluate the effect of laughter yoga on cancer patients undergoing chemotherapy. Sixty-nine participants were included in the study and randomly assigned to either an intervention or control group. The intervention group, consisting of 34 participants, received laughter yoga for four sessions, with a one-week interval between sessions. Each session lasted 20-30 minutes and consisted of 15 steps of laughter yoga performed consecutively. The duration of each laugh was approximately 30 to 45 seconds. Researchers who had completed a laughter yoga training course from a qualified laughter yoga instructor provided the intervention. Participants were supervised during each session, and groups comprising 8, 12, and 14 cancer patients were held. The intervention was introduced before chemotherapy, following a pre-determined protocol. Before the intervention, there were no significant differences between the groups regarding demographic and disease-related characteristics.

The study aimed to evaluate the effects of laughter yoga on various health-related outcomes, including depression, mental well-being, and health-related quality of life. Results showed significant improvements in several domains in the intervention group. Emotional functioning improved by 12.99 ± 10.49, physical functioning improved by 0.78 ± 6.08, and role functioning improved by 3.43 ± 7.97. The intervention group also showed a decrease in fatigue by 8.82 ± 22.01, pain by 8.33 ± 11.78, and sleep disturbance by 15.68 ± 18.77. Moreover, the intervention group's global health and quality of life improved by 6.37 ± 5.04. All the observed changes were statistically significant (p<0.05).

The study also assessed psychological outcomes and revealed that the intervention group had significantly lower post-test depression scores (7.50 ± 6.04) than the control group (12.54 ± 7.53) with a p-value of less than 0.005. Furthermore, a within-group comparison showed a significant decrease in depression scores in the intervention group (p<0.001). The mental well-being of the intervention group, as measured by the Warwick-Edinburgh Mental Well-being Scale (WEMWBS), exhibited significant improvements. Specifically, the mean post-test WEMWBS score in the intervention group (50.0 ± 8.9) was significantly higher than in the control group (47.9 ± 10.4, p=0.004). Repeated measures ANOVA confirmed a significant increase in the WEMWBS scores post-intervention (p<0.001).

In a randomized controlled trial conducted by Han et al., 84 patients undergoing chemotherapy for lung cancer were assigned to either a control or laughter yoga group, with 42 patients in each group [24]. The study aimed to assess the impact of laughter yoga on perceived stress, positive psychological capital, and exercise capacity, with baseline assessments carried out prior to the intervention. The results indicated that patients who received laughter yoga intervention demonstrated significantly higher scores in positive psychological capital (p<0.01, Cohen's d=0.692) and exercise capacity (p<0.01, Cohen's d=0.659) compared to those in the control group. Moreover, discernible differences were observed in perceived stress (p<0.01, Cohen's d=1.087) between the two groups.

Morishima et al. conducted an open-label randomized controlled trial in which 56 cancer patients were randomly assigned to either an intervention group (laughter therapy) or a control group (no laughter therapy) [25]. Each participant in the intervention group underwent a laughter therapy session once every two weeks for seven weeks (a total of four sessions). Each session involved a laughter yoga routine followed by Rakugo or Manzai traditional Japanese verbal comedy performances. We assessed quality of life (QOL) as a secondary endpoint using the European Organisation for Research and Treatment of Cancer Quality of Life Questionnaire Core 30 (EORTC QLQ-C30). The questionnaire was completed at baseline (week 0) and in weeks three and seven. Questionnaire completion rates were high (>90%), with similar QOL scores reported at baseline in both groups. The mixed-effects models showed that the intervention group had significantly better cognitive function and less pain than the control group for a short period.

In their study, Farifteh et al. randomized 37 cancer patients into two groups [26]. The experimental group received 20 minutes of laughter yoga immediately before chemotherapy to evaluate their stress levels. The patients completed a questionnaire called QSC-R23, which is used to assess stress in cancer patients. The collected data was analyzed using a multi-variable covariance analysis test. The results indicated that the intervention effectively reduced stress levels in the experimental group in three sub-scales: psych-physical complaints, fear, information defect, and the total stress score.

Discussion

The combined analysis of data from several studies conducted on Iranian cancer patients undergoing chemotherapy, such as those by Namazania et al., Mohajer et al., and Farifteh et al., provides a comprehensive understanding of the multifaceted benefits of laughter yoga. These studies collectively indicate that laughter yoga can effectively address the physical and psychological aspects of cancer treatment side effects. Upon synthesizing the outcomes of the reviewed studies, we observed that most of the studies used statistical tests such as t-tests, ANOVA, and Cohen's d to assess the impact of laughter yoga interventions. While our review does not introduce novel statistical analyses, understanding the methods employed across these studies is crucial for interpreting their findings. 

Namazania et al.'s study found that laughter yoga significantly improves health-related quality of life (HRQOL) domains, including emotional and physical functioning, fatigue, pain, sleep disturbance, and overall global health [23]. Mohajer et al.'s research documented a notable reduction in depression levels, highlighting laughter yoga's potential to alleviate mood disorders associated with cancer treatments [22]. The 2019 study by Namazania demonstrated significant enhancements in mental well-being, further emphasizing the psychological support laughter yoga can provide [21].

Farifteh et al. focused on the impact of laughter yoga on stress levels in cancer patients immediately before undergoing chemotherapy [26]. Their findings showed a significant stress reduction, suggesting that laughter yoga could serve as an effective intervention to prepare patients for the rigors of chemotherapy, potentially improving their overall treatment tolerance and outcomes.

No adverse events were reported across all studies, confirming the safety of laughter yoga as a complementary therapy in cancer care. High compliance rates further underscore its feasibility and acceptability among patients. These studies collectively argue for including laughter yoga in holistic cancer care programs. By significantly improving physical symptoms and psychological resilience, laughter yoga can enhance the quality of life and potentially impact cancer patients' overall health outcomes. The evidence supports its integration as a regular supportive care option for chemotherapy patients as part of the preparatory regimen to manage stress and improve patient outcomes.

While the results of these RCTs show promise when integrated, we acknowledge several limitations that may affect the interpretation and applicability of the findings. Among these are small sample sizes, heterogeneity of intervention protocols, and short duration of follow-up periods. These factors, in turn, constrain the generalizability of the results and may introduce biases that could impede a clear understanding of laughter yoga's efficacy. Therefore, future research should aim to replicate these findings in larger, more diverse populations with extended follow-up to better understand the long-term benefits and potential mechanisms of laughter yoga in cancer care.

## Conclusions

The body of evidence reviewed indicates that laughter yoga is a highly promising complementary therapy for enhancing cancer patients' psychological and physical well-being. Integrating laughter yoga into standard oncological care protocols has the potential to significantly improve patient's quality of life, particularly during the physically and psychologically taxing periods of chemotherapy. Such practices alleviate treatment-related distress and contribute to overall patient well-being, emphasizing their importance in holistic cancer care strategies. Despite the promising findings, further rigorous research is needed to substantiate these initial results and clarify the optimal timing and context for these interventions to ensure maximum effectiveness. Future studies should focus on detailing the timings of interventions relative to chemotherapy sessions and expanding the diversity of participants to enrich the robustness and applicability of the results across broader oncology practices. By doing so, healthcare providers can better understand how and when to implement such complementary therapies to enhance the therapeutic landscape for cancer patients.
